# Correction: Cost-effectiveness of pulse oximetry and integrated management of childhood illness for diagnosing severe pneumonia

**DOI:** 10.1371/journal.pgph.0004013

**Published:** 2024-11-26

**Authors:** The PLOS GLOBAL PUBLIC HEALTH Staff

An additional affiliation is missing for the second author. Eskindir Loha is also affiliated with the Centre for International Health, University of Bergen, Bergen, Norway and the Chr. Michelsen Institute, Bergen, Norway.

The publisher apologizes for the error.
