# Supplementary material for: Cost-effectiveness of pulse oximetry and integrated management of childhood illness for diagnosing severe pneumonia
Source: PLOS Glob Public Health. 2022 Jul 28;2(7):e0000757. doi: 10.1371/journal.pgph.0000757 (PMC10021260; doi:10.1371/journal.pgph.0000757)
Supplement: Attachment — Submitted filename: Response to Reviewers.docx [file pgph.0000757.s001.docx]

**Date: May 05, 2022**

**To Editor**

PGPH-D-22-00195

“Cost-effectiveness of Pulse oximetry and integrated management of childhood illness for diagnosing severe Pneumonia”

**Dear Editor**

We are grateful for the critical and valuable comments. Below we provide a point-by-point response to each of the reviewers. All the changes made are indicated with track changes in the “*Revised manuscript with Track Changes*”, document.

**Journal requirement**

1. Please ensure you have included the registration number for the clinical trial referenced in the manuscript.

**Response**: We have included the trial registration number and the web address in the revised manuscript (page 5),

2. Please amend your Financial Disclosure statement. If you did not receive any funding for this study, please simply state: “The authors received no specific funding for this work.”

**Response**: The financial disclosure statement is now amended in the “Submission system.” We do not add this statement in the manuscript because we do not get the place of “Funding” section in the formatting guideline.

3. Please provide separate figure files in .tif or .eps format only and ensure that all files are under our size limit of 20MB.

**Response**: We have changed all the figures in “tif” files and all the files are below 20MB in size.

**Reviewer 1 comment**

**Comment** **1**:  The authors provide a very well written economic evaluation of a randomized controlled trial examining pulse oximetry and integrated management of childhood illness for diagnosing severe pneumonia. While there are a few places the manuscript could be strengthened, this is as close as I’ve come in recent memory to suggesting a paper for publication without any revisions. I have only minor suggestions to improve the manuscript that should be addressed before acceptance. They are as follows:

**Comment** 2: Could use some copy editing to tighten up the grammar and English in a few places, but overall very clearly and thoughtfully written manuscript.

**Response: Comment accepted and we have tried to tighten up the grammar and English.**

**Comment** 3: A clarification is necessary on the final row of Table 1. Is transportation per kilometer correct, if applied as a unit cost? Does this assume that the average unit cost of transport per patient is only for 1 km?

**Response**: In the last row of table 1 shows the cost that individuals are expected to pay (tariffs) for transportation per kilometre. Then to get total cost of transportation for patient we multiplied the total distance travelled by the unit cost. However, the average cost is the total cost of transportation divided by the number of diagnosed patients. Not all patients travelled the same distance from their home to access medical care. Therefore the average cost is the cost that patient on average spent for the distance they travelled to access medical care. The estimation of average cost was described in the manuscript.

**Comment 4:** Page 7 line 141. The authors mention an interest rate on the pulse oximeter. Do they mean a discount rate? If so, should provide justification for the interest rate of 7% when 3% is the industry standard. Otherwise, please explain the need for an interest rate… this is unclear.

**Response**: We estimated the average annual cost of pulse oximetry, which has an expected product life of two years. According to Drummond et al 2015, an interest rate of 7% is recommended for costs that has two years life product (reference number “23”, see page 8, methods section, lines 169-170), and therefore this was used in the baseline scenario. A 3% discount rate is used in sensitivity analysis to account for future uncertainty when the time horizon is greater than 1 year. The discount rate used for sensitivity analysis and the interest rate used to annualized capital cost are different.

**Comment** 5: - Page 7 line 149. Are all relevant health workers for this kind of intervention mid-level health workers? Is there a possible upper range of salary reimbursement for some medical professionals that is perhaps not captured in the analysis? Would be good to clarify in the text.

**Response**: Yes, all the health workers who are working in health centres are those with 4 years of medical education background. In Ethiopia it is standard to deploy these mid-level health workers (non-clinicians) in health centres for preventive and basic curative cares. As we described in our published trial paper the mid-level health workers are health officers and nurses who have the same duration of background medical education and both professional categories have a Bachelor of Science degree and their salaries are the same in Ethiopia. Texts clarifying this issue have now been added in the manuscript as follows: “*All the mid-level health workers in the study area are Bachelor of Science degree holder with four years medical education background and their salary is the same.*” (see page 8, methods section, lines 179-181).

**Comment** 6: Figures need to be properly labeled. Not all figures have a full description in the text (see Fig2, especially -not all readers will be familiar with a Tornado diagram).

**Response**: Thank you for this comment, we have now added a description for the figure (see page 14, results section, line 284).

**Comment** 7: Lines 329 - 333 (p16-7) It does seem to be a problem that the facility space itself wasn’t formally added to the cost estimate (even if the facilities already exist, some rental costs, utilities, or other amortized capital costs for the space should be provided to the overall estimate - its not correct to say that just because a new building doesn’t need to be built that the use of the space doesn’t incurr cost). In this case, omitting a cost category of this nature would likely lead to an overestimate of the cost effectivness of either program, but have little to no change in the ICER. That said, this seems like a minimal concern because the facilities already exist, and the program is imbedded in large multiuse facilities. For this reason, space is unlikely to be even a moderate cost driver. Tacit recognition of the absence of these costs is sufficient, but the justification and likely impact on the cost effectivness estimates needs to be reworded.

**Response**: We totally accept the comments of the reviewer. We therefore amended the texts in the manuscript as follows: “*Third, the capital building costs was not included in the cost estimates. However, we expect that improved diagnostics with pulse oximetry may not have a huge impact on capital facility costs compared to the standard intervention assessed in the trial. Therefore it is unlikely, the exclusion of building cost, to have substantial impact on ICER.*” (see pages 18; Discussion section, lines 369-373).

A few additional unnecessary comments to address, but ones you may find worthwhile to consider:

**Comment 1**: I understand the conversation around not including ‘final’ health measures. There could be a back-of-the-envelope calcualtion you could give to estimate the number of cases of severe pneumonia that lead to child mortality, though this is unnecessary. In either case, I don’t think this is enough to overtake the underestimate in cost effectivnesss you face by having excluded opportunity costs of care from the patient perspective however, so I dont see this as a substantial limitation of the paper.

**Response:** We agree with the comment. The trial was conducted in rural primary health centres where diagnosis of severe pneumonia is based on signs and symptoms. There are two types of outcome measures in economic evaluations namely final outcome and intermediate outcome. Therefore we mentioned this as potential limitation of our paper thinking that it would have been better to include the final outcome.

**Comment**: 2 - Given the paucity of good primary cost data on these kinds of studies, unless you are planning a separate costing exercise, you should consider a table breakdown of the average cost at each of the facilities in the study, and provide some characteristics of these facilities to help explain how costs differ by study setting. This would be a major value adds to the field and would only strengthen the overall publication.

**Response**: We agree with the comment and provide the average cost at each facility for both the intervention and control arm separately (see table below). As we can see from the table the average costs of facilities in the intervention arm are large than the facilities in the control arm. This is what is expected, because of the incremental costs due to pulse oximetry in the intervention arm. There is no substantial difference in average cost among the health facilities in both arms. As we stated in our published trial article the characteristics of the health facilities are the same. The human powers, availability of resources, the health systems for management of childhood illness are the same as these characteristics are settled as standard by the government. All the health facilities are primary health centres all located in rural area. The cost of management of severe pneumonia is the same across the facilities as stated by the government. The only difference across the facilities in both arms is the distance between the location of the health facilities and the residential area of the patients. As a result the cost spent for transportation might be different. But this difference does not have substantial effect on average cost at each facility. Therefore, we haven’t seen any substantial difference at each facility that is attributable to their specific characteristics.

| **Intervention group** | | **control group** | |
| --- | --- | --- | --- |
| **Name of Health centres** | **Average cost (range) USD** | **Name of Health centres** | **Average cost (range) USD** |
| Adado | 24.30 (23.88-24.69) | Kelecha | 21.66 (21.66-21.66) |
| Andida | 23.05 (22.77-23.37) | Chito | 16.88 (14.88-20.78) |
| Bulle | 25.15 (24.79-27.31) | Edido | 19.45 (18.08-22.19) |
| Chelelektu | 29.10 (28.52-30.44) | Kedida | 24.36 (24.16-24.56) |
| Chichu | 22.42 (22.36-22.67) | Mekonisa | 19.55 (19.36-19.74) |
| Gedeb | 30.20 (29.73-30.74) | Moralayo | 19.28 (13.28-20.56) |
| Gerse | 27.81 (27.51-29.23) | Odaya'a | 18.13 (18.13-18.13) |
| Dilla | 22.27 (22.26-22.26) | Sokicha | 16.88 (11.68-16.56) |
| HaseHaro | 24.64 (24.28-25.19) | Udo | 15.68 (15.68-15.68) |
| Laba Reji | 32.02 (30.84-32.66) | Weka Dibitu | 16.21 (15.68-17.25) |
| Shifo | 30.10 (29.33-30.44) | Wonago | 16.32 (12.07-19.97) |
| Tumticha | 28.88 (28.32-29.20) | Yirgacheffe | 20.55 (15.10-22.75) |

**Comment 3**: - Providing any additional detail of the sample of participants out of pocket costs would also be helpful in framing the relevant population under study. This data can probably be cross referenced from the RCT, but a recitation of those characteristics here would add value.

**Response: Yes, estimates of out-of-pocket payments according to different categories of caregivers’ welfare level would be important. As we describe in result section of the published trial there is no significant difference among the caregivers’ wealth index. The cost for health care seeking is equal for all sick children. The main driving costs for the caregivers in both arms are cost of drugs and supplies, and transportation costs. The potential impact of these out-off-pocket payments on caregivers was discussed in the manuscript (see pages 16, 17, lines 330-350).**

**Reviewer 2 comments:**

**Comment 1:** This paper has the potential to make an important contribution to the literature. The authors do define a specific objective to compare the cost-effectiveness of combined use of pulse oximetry with standard IMCI guideline compared with the standard IMCI guidelines alone to improve diagnostic precision of severe childhood pneumonia in rural Ethiopia. In the methods section, they narrow the scope a bit by focusing on opportunity costs of combined use of Pulse oximetry and the standard IMCI guideline versus the standard IMCI guideline in an Ethiopian rural setting. This paper would benefit from a careful explanation of the methods using best practice and guidance from the literature on CEA of diagnostic approaches. As written, I can’t really assess whether what the authors have done is a cost-consequence analysis or truly a cost-effectiveness analysis. It may be useful to be more explicit and systematic about the methods throughout the paper. For example, justify your choice of effectiveness, excluding all benefits of improved dx in intro, be clear in methods why some treatment costs included, but averted treatment costs not included, and add any caveats to this approach in discussion). It may be the authors want to focus on cost-effectiveness of an approach to detect pneumonia, rather than cost effectiveness of diagnosis, which has implications for the analysis. For instance, CEA of diagnostic tests/approaches are stronger when looking at cases ‘accurately’ diagnosed. This information is not available. In addition, the authors do not explicitly look at a health outcome per se, which is also a hallmark of cost effectiveness analyses, if not looking at diagnostic accuracy. I understand the authors saw this as a caveat, but it is worth including these issues in the methods, rather than a caveat.

**Response:** We agree with the comment given by the reviewer regarding the justification of the choice of health outcome and inclusion/exclusion of treatment costs. Because of the context of the health system of the settings, we used severe pneumonia detected as primary outcome (intermediate health outcome). We assume that correct diagnosis of severe pneumonia using the intervention could lead to correct treatment decision making, even though there are false positive and false negative cases of severe pneumonia, which we were unable to estimate the accuracy of the trial interventions. Because of lack of availability of reference standard diagnostic tools in these rural part of the study settings to estimate the diagnostic accuracy of the interventions used in this study, we did not include the averted treatment costs in the analysis. We include costs related to diagnosis of severe pneumonia and out-of-pocket payments to access medical care. However, since we are looking at incremental cost of pulse oximetry there will be out-of-pocket payment incurred both on health system and patients. For example child diagnosed as severe pneumonia with both IMCI and pulse oximetry has implication on management and incurred cost for drugs, hospital stays and transportations. Because of this we included these costs in our cost-effectiveness analysis. Had pulse oximetry been not used in the settings, those identified severe pneumonia cases might have been missed and do not impose costs related to drugs, hospital stays and transportations on patients.

In sum, we share the concern raised by the reviewer and we add some text to clarifying how we revised our approach of health outcomes and the inclusion and exclusion of some treatment costs in the revised manuscript (see page 6, methods section, lines 139-143 and pages, 9, 10; lines; 206-215) and discuss the implications in the discussion part of the revised manuscript (see pages 17, 18; discussion section, lines; 360-367).

**Introduction**

**Comment: 1** Line 55-57: Feasibility of adding a new technology that requires training in a situation where even a more basic established protocol (IMCI guidelines) can’t be scaled….Pulse oximeter is a medical supply, requires training, maintenance…Implementation of standard IMCI is difficult due to lack of trained staff, lack of drugs and supplies and inconsistent supervision. Can the authors explain how adding additional diagnostic tools and supplies (for oxygen therapy) at added cost addresses/overcomes the health system constraints described and the consequent IMCI ‘know-do’ gap? The justification for this could be stronger in the intro (and the authors may want to revisit this in the discussion).

**Response:** The clinical signs included in IMCI guideline have low sensitivity to detect severe pneumonia. However, pulse oximetry is an objective tool to accurately detect hypoxemia as sign of severe pneumonia in children with cough/difficulty breathing. Our published trial article (Tesfaye et al, 2020) shows that diagnoses of severe childhood pneumonia increased from 4% using the standard IMCI guideline alone to 16% using the combination of pulse oximetry with the standard IMCI guideline. In addition, supplementing IMCI with pulse oximetry needs additional investment of $ 29 to detect one more child with severe pneumonia. Therefore, with two days training of health workers about IMCI and handheld paediatric pulse oximetry is feasible to overcome the gap in IMCI guideline to diagnose severe pneumonia cases. Our trial addresses an important, pragmatic case diagnosis intervention of wide relevance for rural health centres where there is low diagnostic capacity.

**Methods**

**Comment**:1 1. Line 115, Page 5- the health effect is the number of severe pneumonia cases detected—do the authors have any information cases were correctly detected? (from looking at the RCT results, I believe not). Or, if the cases detected were treated with pre-referral drugs, or referred to a primary hospital for treatment? From table 1, it appears patients in both settings did receive oxygen treatment, drugs and IV fluids. Would it be possible to look at improvements in dx and treatment at the health center level?

**Response: We didn’t estimate the number of correctly diagnosed severe pneumonia case for different reasons: 1) reference standard diagnostic tools are not available in health centres and the Ethiopia health policy don’t allow health workers to do chest-x ray or any other bacteriological or imaging technique to diagnose severe pneumonia at health centres. 2) Even in hospitals bacteriological or imaging technique is not a requirement to diagnose severe pneumonia and there is also lack of these tools in the study settings at hospital level. According to the Ethiopian health policy severe cases of pneumonia or any other severe childhood illnesses should be referred to primary hospital for in-patient management. As we mentioned in the manuscript under study setting oxygen therapy is only available at hospital (see page 5, study setting section, lines; 118-119). Severe pneumonia cases were referred to hospital after giving single dose of antibiotics. Therefore health workers at health centre in both arms after diagnosing severe pneumonia cases give first dose of antibiotics at health centres, then immediately refer the child to hospital and the child will get the inpatient management at hospital (oxygen therapy, IV fluids, and drugs). There is 12 percentage point increment in diagnosis of severe pneumonia at health centres due to the interventions. Health workers at health centres didn’t provide treatment for severe pneumonia (except giving first dose of antibiotics) and we are unable to estimate the number of improvement at the health centres level. We only intervene on diagnosis and it is possible for severe pneumonia cases in both arms to be referred to hospital and get the standard in-patient treatment protocol (example; oxygen therapy, and antibiotics).**

**Comments:** 2. Intervention costs from provider and patient perspective—

a. The authors capture treatment costs; however, they do this by capturing the patient out-of-pocket expenses, which include treatment costs (drugs and supplies). Do the authors consider this the full opportunity costs to the patient? Or do these costs also represent the health system costs of treatment at the health center level, since it captures drugs, supplies and a government surcharge of 25% surcharge, which presumably captures personnel and overhead costs. Suggest the authors be more explicit about this in methods, and revise, line 133 to read, “We therefore included treatment costs as part of patient costs (Table 10).”
i. If these treatment costs represent both patient cost and health system costs, you may want to justify by indicating you captured treatment costs on the patient side to avoid double counting.

**Response**: **i** According to the comment we now added text clarifying this issue as follows: “*Patients pay out-of-pocket for drugs and supplies from government health facilities, and the government applies a 25% surcharge to these items. Costs for drugs and supplies represent both the health system costs and patient costs. Therefore we include cost for drugs and supplies on patient side to avoid double counting.*”(see page, 7, method section, lines, 156-158).

ii. Are these intended to represent opportunity costs to the patient and health system, or just to patient? If just patient opportunity costs, please indicate more clearly this is your measure of patient opportunity costs.

**Response: ii** For the reason we mentioned above and because of the out-of-pocket payment for drugs and supplies by the patient would have been used for other activities by the patient. We consider the drugs and supplies cost as patient opportunity cost for our study. We now included this text in the revised manuscript (see page 7, method section, lines 158-159).

**Comment** **b**. Are there any averted costs associated with untreated severe pneumonia, such as hospitalization or longer hospitalization stays? If yes, and these are not captured in this analysis, suggest being explicit that this analysis does not include averted health system and patient costs from improved dx.

**Response: Our analysis didn’t include the averted costs due to improved diagnosis of severe pneumonia cases because we are unable to estimate the accuracy of the diagnostic interventions. We accept the comment and add statement in the manuscript to explicitly justify our approach** (see page 6, method section, lines; 139-143).

**Comment**: C. Related to averted costs due to improved dx, it does appear that this analysis does not look at treatment outcomes and net costs (intervention costs plus incurred health system treatment costs minus health system and patient medical costs averted), however, (1) it is a little confusing why the authors are capturing the treatment (partial? Pre-referral?) costs to the patient at the health center level; and (2) why they don’t include full range of benefits from improved dx. It would be useful for the authors to clarify this in intro, methods, and/or explicitly note this in the discussion.

**Response (1):** Health workers at health centres should refer severe pneumonia cases to hospital after giving single dose of antibiotic. The cost for this single dose antibiotic is covered by patient-out-of-pocket payment. Therefore, we include the cost of pre-referral drugs at health centres. But cost of drugs, oxygen therapy, IV fluids and supplies costs are included at hospital level, because patients are getting these treatments at hospital level. Based on the comment given by the reviewer we included texts explaining cost included for pre-referral drugs at health centres level and costs for full inpatient management including costs for drugs, hospital stays and supplies, and oxygen therapy included at hospital (see page 7, method section, lines 159-162).

**Response** **(2)** It would have been better to include health benefits from improved diagnosis of severe pneumonia. One of the benefits from the improved diagnosis of severe pneumonia is life years gained or deaths averted due to improved diagnosis. However, because of logistic constraint we recruit and conducted the study for 8 months and as a result we didn’t capture sufficient number of deaths from severe pneumonia. For example during the study period there were only 4 deaths. We accept the comment and we now clearly indicate this in the manuscript (see pages 9, 10 method section, lines; 206-215).

**Comment**: **d**. Page 7. Line 151. First indicate how total cost was calculated. This isn’t clear. You estimate costs for each arm (intervention and control) using input costs shown in table 1, but how do you get a total cost for each arm, and then describe the three three average costs (all pneumonia, severe, non-severe)?

**Response:** We accept the comment and explain how the costs were derived (see pages 8, 9; methods section, lines: 182-195).

**Comment**: **e**. Page 9, table 2—how did the authors pick the min and max estimates for costs?

**Response:** We estimated the mean for cost and proportion of diagnosed severe pneumonia with the 95% CI and the minimum and maximum values of the 95% confidence interval were used for both outcome and cost. (page 11, methods section, lines 238-239).

**3. Cost-effectiveness model**

**Comment**: **a**. The authors develop a model that compares the opportunity cost of combined use of Pulse oximetry and the standard IMCI guideline versus the standard IMCI guideline in an Ethiopian rural setting. I find the wording of this strange, because they are comparing approaches on an intermediate outcome, i.e. the measure of effectiveness is the proportion of children diagnosed with severe pneumonia in the intervention and control. Perhaps just refine the statement on what they are comparing and be more explicit up front about comparing opportunity cost and intermediate outcomes.

**Response:** We use the proportion of severe pneumonia cases detected (intermediate outcome). According to the comment by the reviewer we included the following sentence in the revised manuscript: *“The model compares the opportunity cost and the proportion of severe pneumonia cases detected using combined use of Pulse oximetry and the standard IMCI guideline versus the standard IMCI guideline in an Ethiopian rural setting.”* (see page 9, method section, lines; 202-204).

**Comment b**. A cost effectiveness analysis ought to evaluate what occurs after the diagnosis, and fully evaluate the health and cost outcomes for each diagnosis. This could be an improvement in diagnostic accuracy, or changes in health outcomes, resulting from diagnosis and treatment.

**i**. The authors should explain why additional benefits (i.e. improved health outcomes and averted costs to the health system and patient—averted medical costs, averted health system expenditures), from improved dx, were not included.

**Response**: In this rural part of Ethiopia, where reference standard diagnostic accuracy test is lacking, it is difficult to get all aspects of improved health benefits from diagnosis of severe pneumonia. We agree with the reviewer’s comment. We conducted the study for 8 months which is short period to estimate the final health benefits such as life years gained or mortality averted. During the study period there were only 4 deaths. We accept the comment and add statement explaining what the cost-effectiveness model includes/addresses (see methods section, page 6 lines 139-144 and page 9; lines 206-215).

**Comment: ii**. Can the authors justify why they chose the measure of effectiveness they did?

**Response:** It would have been better to use final end points as a result of improved diagnosis, but because of the context of the study settings and lack of logistics for diagnostic accuracy test we choose the proportion of diagnosed severe pneumonia cases as measure of effectiveness. But we accept the recommendation made by the reviewer below and amend accordingly in the revised manuscript (see page 9, lines 206-215).

**Comment: c.** To the points above, I recommend that the authors more clearly describe their cost-effectiveness model and better define the incremental cost effectiveness ratio up front, and not leave it to the discussion section and the caveat. This is an important issue. I don’t disagree with what the authors say, but one may also wonder, if pneumonia is a well-established leading cause of death and they have information on numbers treated and their costs, then why not model that out? Explain up front, why this approach was not done, or feasible. And to support your approach, reference Drummond et al in the methods section.

**Response:** We totally agree with the recommendation and we now added the following statement in the revised manuscript: “*We conducted the study in rural part of Ethiopia where non-clinicians are responsible for diagnosis of illness. Once a child is identified with severe pneumonia the health workers should refer the child to hospital where clinicians are responsible for child care. As a result we didn’t include the final health outcome at hospital level in the cost-effectiveness model. We accept the comment of the reviewer and we provide explanation for comments raised under comment number “i” and “ii” in the revised manuscript as follows: “In this study we didn’t analyse improved health outcomes and averted costs to the health system and patient, averted medical costs, averted health system expenditures from improved diagnosis at health centre levels. To use mortality averted or life years gained due to interventions as final end point is not feasible, due to the fact that we conducted the study for short period of time (8 moths). During this period there were only four deaths.”*(see page 9, methods section, lines; 206-215).

**Comment**: **i**. Perhaps the authors can review this useful check list to justify their approach. Kip, Michelle MA, et al. "Toward alignment in the reporting of economic evaluations of diagnostic tests and biomarkers: the AGREEDT checklist." Medical decision making 38.7 (2018): 778-788.

**Response:** We thank the reviewer to share this article.

**Comment**: **d**. Table 2. Input parameters. Do the costs of diagnosed severe and non-severe pneumonia include the cost of treatment at the health center? If so, shouldn’t the input parameter read, cost of dx and pre-referral treatment of severe pneumonia…’ if and when that is the case?

**Response**: Yes cost for diagnosing severe pneumonia includes cost of pre-referral drugs at health centres and drugs taken at hospital. When we say “cost of diagnosed severe pneumonia” we mean that these costs include all the costs presented in table 1 of the manuscript. Severe pneumonia is not treated at health centre level, except giving first dose of antibiotics. The cost of diagnosis of severe pneumonia in table 2 includes the costs of all the items in table 1 including the cost of pre-referral drugs at health centre level. For analysis purpose we sum up the cost of pre-referral drugs at health centre and cost of treatment at hospital level together. But the costs of diagnosis of non-severe pneumonia include costs at health centre level.

**Comment**: **e**. How are Table 2 and 3 related? Is Table 3 JUST diagnostic costs, without treatment? If treatment costs aren’t used in the ICER, I’m not sure why they are included in the cost analysis.

**Response:** Table 1 in the manuscript shows the input parameters for the cost-effectiveness analysis. The cost diagnosis in table 2 includes all the items mentioned in table 1. Treatment costs are consequence costs as a result of diagnosis using the interventions. Unless the child is diagnosed for severe pneumonia, costs for drugs, supplies, transportation, hospital stays, and oxygen therapy wouldn’t be spent by the patients. According to Drummond et al, 2015, if the interventions have consequence costs, those costs should be included in the analysis. We presented the input parameters in table 2 as costs for diagnosis (all items in table 1), and proportion of case detected. We did this to use them in the TreeAge software. Because, in TreeAge software, as shown in Fig.1 of the manuscript, there is one path (nodes) for costs, and another node for cases detected. That is why we combined all the items’ cost in table 1 as “diagnostic cost” and presented them combined in table 2. Table 2 is useful for readers who want to replicate the cost-effectiveness analysis. In table 3 we want to show which input parameters in table 1 constitute the largest and smallest share of the total costs.

**Comment**: **f**. Pg. 10, Line 198-199- What was the time horizon for the actual RCT study? The total and average costs have been calculated from a sample over a given period (i.e. one year or two years?)- I believe this is the correct time horizon, if they are calculating total costs and dividing by all diagnosed cases in intervention and control.

**Response:** We collected data over 8 month period. Yes we accept the comment than the time horizon is the overall data collection period. The average cost was calculated by dividing the total cost by all diagnosed cases.

**Results and Discussion**

**Comment**: **1**. Since effectiveness estimates are the result of another study (referenced), perhaps just refer back to Table 2, and do not make this a result for this study. It is an input into your model.

**Response:** Yes, the parameters in table 2 are result of the trial we published. We presented the input parameter table 2 as part of the methods not as part of our result. According to the comment we now cite reference to the table (see pages 9, 10 table 2).

**Comment**: **2**. Could you add a new table to show total costs, numbers of cases and average costs for (1) Intervention dx severe (2) intervention non-severe; (3) Control dx severe, and (4) control non-severe –so four columns, and this would correspond to the input costs shown in Table 2, but the reader can understand better how they were derived. You could include information from previous RCT study shown in lines 205 and 206, p. 10 in this table, as it is a given input into your analysis, not a result.

**Response:** Below we provided the new table according to the comment and add it into the revised manuscript as “Table 3” (see page 13, Table 3).

**Table 3 Total and average cost for diagnosed pneumonia cases.**

| Diagnostic alternatives | Severe pneumonia | | | non-severe pneumonia | | |
| --- | --- | --- | --- | --- | --- | --- |
|  | Number diagnosed | Total cost (USD) | Average cost (USD) | Number diagnosed | Total cost (USD) | Average cost (USD) |
| Standard IMCI alone | 34 | 611.30 | 17.98 | 842 | 1800.17 | 2.14 |
| Standard IMCI and pulse oximetry combined | 148 | 3809.24 | 25.74 | 780 | 2794.45 | 3.58 |

**Comment**: **3**. I don’t understand the relationship between Table 1 and 3. If in methods you describe how you calculate total cost, this may become clearer.

**Response:** Table 1 presents the unit cost of each item (initial cost). Table 3 presents the average cost of each items presented in table 1 and their sharing. But now we amend table 3 according to the comment (presenting cost shares from the total costs) and table 3 changed to table 4 because we added the above new table as “Table 3” (see page 13; Table 3 and Table 4, Result section).

**Comment**: **a**. I would think Table 3 would present a summary and disaggregated cost categories for total costs, corresponding to lines 212 and 213, but these look like average unit costs again. It makes more sense to me to look at cost shares of the total costs disaggregated by cost categories.

**Response**: According to the comment the cost shares are now indicated using total costs and “Table 3” is changed to “Table 4” (see page 13, Table 4).

**Comment**: **4**. Table 4 and CE results seem more like a cost analysis to me. In the discussion, the authors note that comparisons are difficult to make, in part b/c other similar studies do extend the analysis out to cases treated or DALYs averted. I think this paper could be reframed as a cost consequence analysis and would be an excellent complement to the published RCT. Especially since much of the discussion focuses on costs.

**Response:** We accept the comment. But for the reasons we mentioned above and the context of the study settings we didn’t use final health outcome and instead we use the proportion of severe pneumonia case detected as outcome (intermediate outcome) according to Drummond et al fourth edition, 2015, pages 5-7 and Gray et al 2nd edition, 2012, page 9.” The outcome we used in this study is only in the context of rural settings in Ethiopia. But we are not sure if the reviewer is suggesting for the title to be reframed as “cost consequence…..” if that is the case we welcome the comment.

**Minor**

**Comment**: **1**. Line 141, what are ‘initial costs’ for pulse oximeter?

**Response:** It is USD 149.81 (see pages 7, 8; table 1)

**Comment**: **2** Add year of currency to Table 1

**Response:** We accept the comment and added the year of currency to table 1 (see pages 7, 8, table 1).

**Comment**: 3. Add currency and year to Table 3 and indicate these are costs for diagnosing severe pneumonia.

**Response:** We accept the comment and currency and year are now added and the title of the table is now became “Table 4” (see page 13, Table 4).

**Comment**: **4** Since the approach is incremental to the existing IMCI health program, I think the authors can omit lines 330 to 333—unless the improvements used more space/bldg., this would net out to be zero.

**Response:** The other reviewer of this paper comments to justify the reason for omitting the costs and reworded the potential/likely impact on cost-effectiveness result. Therefore, we reworded the previous texts explaining the potential impact on the result to address the reviewer comment.
